# An Energy-Efficient Skyline Query for Massively Multidimensional Sensing Data

**DOI:** 10.3390/s16010083

**Published:** 2016-01-09

**Authors:** Yan Wang, Wei Wei, Qingxu Deng, Wei Liu, Houbing Song

**Affiliations:** 1 School of Information Science and Engineering, Northeastern University, Shenyang 110819, China; wang_yan@lnu.edu.cn (Y.W.); liuwei-neu@hotmail.com (W.L.); 2 School of Information, Liaoning University, Shenyang 110036, China; 3 School of Computer Science and Engineering, Xi’an University of Technology, Xi’an 710048, China; weiwei@xaut.edu.cn; 4 Department of Electrical and Computer Engineering, West Virginia University, Montgomery, WV 25136, USA; Houbing.Song@mail.wvu.edu

**Keywords:** CPS, WSN, skyline query, energy-efficient, node cut

## Abstract

Cyber physical systems (CPS) sense the environment based on wireless sensor networks. The sensing data of such systems present the characteristics of massiveness and multi-dimensionality. As one of the major monitoring methods used in in safe production monitoring and disaster early-warning applications, skyline query algorithms are extensively adopted for multiple-objective decision analysis of these sensing data. With the expansion of network sizes, the amount of sensing data increases sharply. Then, how to improve the query efficiency of skyline query algorithms and reduce the transmission energy consumption become pressing and difficult to accomplish issues. Therefore, this paper proposes a new energy-efficient skyline query method for massively multidimensional sensing data. First, the method uses a node cut strategy to dynamically generate filtering tuples with little computational overhead when collecting query results instead of issuing queries with filters. It can judge the domination relationship among different nodes, remove the detected data sets of dominated nodes that are irrelevant to the query, modify the query path dynamically, and reduce the data comparison and computational overhead. The efficient dynamic filter generated by this strategy uses little non-skyline data transmission in the network, and the transmission distance is very short. Second, our method also employs the tuple-cutting strategy inside the node and generates the local cutting tuples by the sub-tree with the node itself as the root node, which will be used to cut the detected data within the nodes of the sub-tree. Therefore, it can further control the non-skyline data uploading. A large number of experimental results show that our method can quickly return an overview of the monitored area and reduce the communication overhead. Additionally, it can shorten the response time and improve the efficiency of the query.

## 1. Introduction

The cyber physical system (CPS) senses the environment based on wireless sensor networks (WSNs). Currently, there are many CPS applications, such as smart grid and ice-disaster monitoring systems and mining safety monitoring and control systems. In these monitoring systems, users must know the situation of the entire monitoring area in time to provide early warnings about the possibility of failure or danger [1,2,3]. As one of the major monitoring methods in safe production monitoring and disaster early-warning applications, skyline query algorithms are extensively adopted for the multiple-objective decision analysis of these sensing data [4]. For example, in ice-disaster warning systems, the power-grid transmission lines can easily ice over when there are conditions such as low wind speed, low temperature, and high humidity in a region. Then, the centralized control can send the skyline query of the (speed, temperature, humidity) dimension to the sensor networks that are deployed in power-grid transmission lines and thus determine the presence of iced transmission lines or will-be-iced lines in time. The query scheme can help to prevent wires from icing and make the ice melt within a short time by increasing the temperatures of the wires [5,6,7]. These applications involve an extensive geographical distribution, large-scale WSNs and a large amount of sensing data. In addition, all of the sensing data are massive and multi-dimensional. Thus, reducing the transmission energy consumption is necessary and important [8]. Because these systems have strict requirements on the data validity and the timeliness of the feedback control, they must address the massive data more quickly [9,10] and implement the energy efficient query for the area that is prone to danger or disaster [11,12,13].

Traditional skyline query methods in WSNs have the common features of considering only the domination relationships among the data; thus, the cutting capacity of these algorithms is not large. Although the description of the algorithm is simple (the time complexity is O(n)), the network transmission cost is high. Therefore, they are applied only to the small monitoring systems in which the sensor distribution is denser. The energy available to a sensor node is limited, and the energy consumption of the communications is much greater than that of the computing. Therefore, the key problem to be solved urgently is how to reduce the transmission cost. This paper proposes E2Sky, an Energy-Efficient Skyline query method for massively multidimensional sensing data. This method uses a node cut strategy to dynamically generate filtering tuples that have little computational overhead when collecting query results instead of issuing queries with filters. It can judge the domination relationships among different nodes, cut the detected data set of dominated nodes that are irrelevant to the query, modify the query path dynamically, and reduce the data comparison and computational overhead. The efficient dynamic filter generated by this strategy uses little non-skyline data transmission in the network, and the transmission distance is very short, even if there is non-skyline data transmission. The method also employs the tuple-cutting strategy inside the node and generates the local cutting tuples by the sub-tree that has the node itself as the root node, which will be used to cut the detected data within the nodes of the sub-tree. Therefore, it can further control the uploading of non-skyline data. Through the above strategy, our approach can quickly return an overview of the monitored area, reduce the consumption of the communication, shorten the response time, and improve the query efficiency. Moreover, the approach also has good scalability as the size of the WSN becomes large. The major contributions of this paper are as follows:

- We design a dynamic filter tuple and a local cutting tuple, propose a calculation method of the two types of filter tuples and analyse the effectiveness of the cutting tuple in depth.
- Based on the above dynamic filter tuples and the local cutting tuple, we propose E2Sky, a query recovery mechanism, which adopts node cut and tuple cut inside the node algorithm.
- We design detailed performance evaluation experiments. The experimental results show that the E2Sky algorithm reduces the transmission consumption of the skyline query recovery greatly, with a small calculation overhead.

The remainder of this paper is organized as follows: Section 2 discusses the related work. Section 3 presents the concept and the problem definition of the skyline query. Section 4 describes the query result collection algorithms in E2Sky. The experiments and the performance comparison are discussed in Section 5. Finally, Section 6 concludes this paper with future work proposals.

## 2. Related Work

A traditional skyline query is mainly based on the centralized database. Many algorithms have been proposed in centralized environments. Reference [14] proposes two centralized algorithms, namely, Block Nested Loops (BNL) and Divide-and-Conquer (D&C). BNL compares each point with a list of skyline candidates that are kept in the main memory. Dominated points are pruned after comparison. D&C first divides the data set into several partitions that can fit into memory. Skyline points for each partition are then computed, and the final skyline can be obtained by merging these skyline points. Reference [15] proposes an algorithm that is called sort-filter-skylines (SFS) as a variant of BNL. Reference [16] proposes two progressive processing algorithms, Bitmap and Index. Reference [17] proposes a nearest neighbour (NN) method to process skyline queries progressively. NN recursively searches for the nearest neighbour in the current region (full data space first) and divides the region into smaller sub-regions. The nearest neighbour can be immediately output because it must be a skyline point. Reference [18] improves the NN method by using the idea of branch-and-bound (BBS). It only traverses the R-tree once and processes the entries in ascending order of their minimum distances to the origin of the data space. However, their approaches focus mainly on answering the skyline queries in centralized environments and do not consider the limited computational power of sensor networks. Moreover, these methods increase the transmission overhead because of transferring data from the sensor to the central server.

For skyline queries in wireless sensor networks (WSNs), [19,20,21] propose three types of filter-based skyline query approaches. Of these, the method in [19] is similar to that in [20] but has a different way of choosing the local filter. Both of them broadcast an initial filter to their children from the root and update it at each node to obtain the “best” filter in the leaf node last, which dominates most of the nodes. Then, it is returned to the root with the query results to have a second cut. This process generates large amounts of communication costs in the data gathering. Reference [21] proposes a hierarchical threshold-based approach, MINMAX, whose filter is generated within each sensor. This approach will lead to a large amount of non-global skyline data transmission among the sensor nodes. Recently, a novel approach, FIST [22], is proposed to evaluate the continuous skyline node query in the wireless sensor networks. It installs local or global filters within each sensor to reduce the amount of data that is transferred among the sensor nodes. The global filter is more suitable for small-scale sensor networks, but not for a large-scale network.

References [23,24] have investigated skyline queries based on sliding windows. Reference [23] proposes an algorithm that is based on a tuple filter and a grid filter, which can continuously query the skyline of the sliding window in the WSN. Their approach can filter out non-skyline data in each sensor and finally return the final skyline to the base station to minimize the communication cost in the sensor network. Reference [24] proposes a skyline query that maps the sensing data to a certain space of integers by a mapping function. In particular, a mapped skyline filter (MSF) resides in each sensor node and filters the tuples but has no contribution to the final result. Therefore, the energy consumption is reduced significantly. Determining the sliding window is a problem in these approaches. In [23], a large amount of preparation work must be accomplished before cutting the non-skyline data. In [24], when the tuples are centrally distributed near the actual skylines, the cut efficiency of the middle filter is low, which will generate a larger communications overhead while transforming the non-skyline data in the query result collection.

A cluster-based architecture is designed in SkySensor [25], which collects all of the sensor readings. The attribute that has the smallest value in a detected tuple determines the cluster in which the tuple should be stored. In query processing, this approach can quickly prune the storage nodes that do not need to be visited in storage clusters, and thus, the traffic to the storage nodes is reduced. However, the detected data tuples are stored in a suitable cluster in a distributed manner in this method, which cannot be applied to the WSN in a large-scale WSN.

However, all of the methods mentioned above focus on the dominance relationships in the data, and they do not account for the data set in the sensor nodes. Therefore, these approaches will generate high energy consumption and low query efficiency when applied to the query in a large-scale WSN.

## 3. Problem Definition

In the database, the data skyline of the data sets is comprised of tuples that are not worse than any other tuples in that data set. First, the “good and bad” relationship between two tuples is defined as the domination relationship (as shown in Definition 1). Based on these domination relationships, a skyline query is intended to decrease the amount of non-skyline data and reduce the transmission of useless data in the network.

*Definition 1*: For any two tuples *t_i_* and *t_j_* in a tuple set *T*, the domination relationship between them can be defined as follows: there is a domination relationship if any dimension data on* t_i_* is not worse than the corresponding dimension data on *t_j_*, and at least one dimension data on *t_i_* is better than that on *t*_j_. We can define that *t_i_* dominates *t_j_*, which is denoted as *t_i_* ≺ *t_j_*.

**Figure 1:**
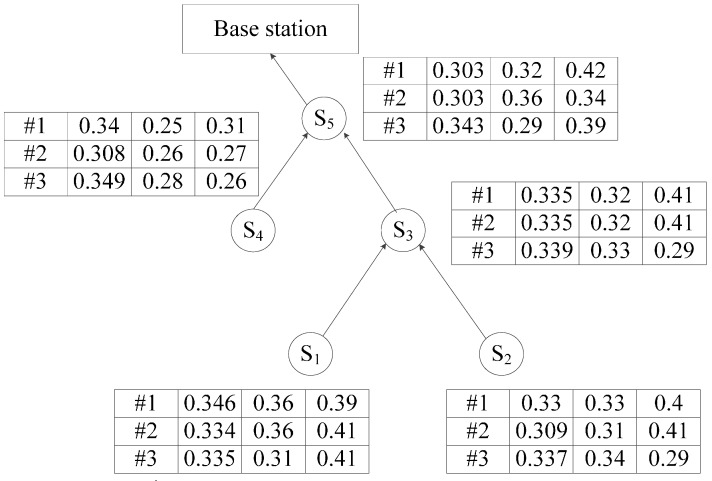
An example of a skyline query.

As shown in Figure 1, *S_1_*, *S_2_*, *S_3_*, *S_4_* and *S_5_* constitute a routing tree, where *S_5_* is the root node, and the detected data of the node in the consecutive three moments are given in the table beside the node. Among them, the first column shows the data acquisition time, and the remaining columns show the detected value of the sensor node. Network computing technology [23] is a skyline query algorithm. First, the routing tree rooted at the base station is built. Second, the leaf nodes compute the local Skyline data and send them to their father nodes. Then, the father node merges them with its own data to compute a new local Skyline result and submit it to its father node. Finally, in this way, the base station will obtain the global skyline results. This arrangement requires that each node is only required to transfer the local skyline of the sub-tree for which that node is the root. Hence, there are 28 tuples transferred in three time steps. The higher the dimensions of the detected data, the less duplicated the data in each node. The network computing method still causes a large amount of useless data transmission in the network. For example, the detected data of node *S_2_* at the second time is (0.309, 0.31, 0.41), which is dominated by the detected data in node *S_4_*. However, it will still be transferred to node *S_5_*. Thus, it is important to research Skyline query algorithms that may have stronger cut efficiency to avoid such unnecessary data transmission and to accelerate the speed of returning the query results.

**Figure 2:**
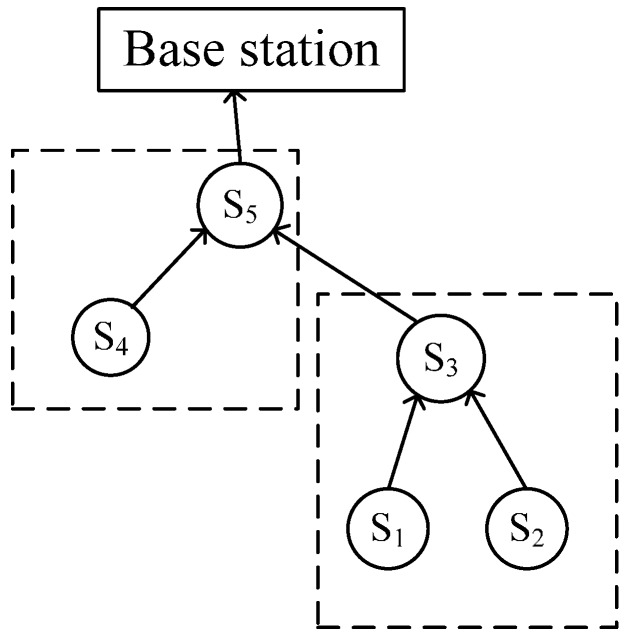
Diagram of the node domination relationship.

The Energy-Efficient Skyline Query method for massively multidimensional sensing data (E2SQ) in this paper is based on the establishment of the routing tree, which sets the base station as the root node. First, the system initializes the data structure of each node through the query result collection algorithm. The *qdata* table and *filter_tuple* table of each sensor node are set up (see Algorithm 1 in Section 4.1). The query processing system will compare the skyline of the sub-trees, which are shown as the dashed area in Figure 2, and find a certain node set. Then, it will decrease the number of nodes that are not in the node set from the query route by the node cut algorithm (see Algorithm 2 in Section 4.2); Second, the system finds the local cut tuple by the cutting tuple extraction algorithm (see Algorithm 3 in Section 4.3), and then it cuts the query data of the remnant nodes in the query route tree according to the local cut tuple. Finally, the system collects the skyline data and sends them to the root node. The query result collection mechanism can improve the cutting efficiency of the skyline query and reduce the communication cost of the non-skyline data within the network, which prolongs the network’s lifetime.

## 4. Query Result Collection Algorithms in E2Sky

### 4.1. Initialization of the Query Result Collection

#### 4.1.1. Design of the Data Structure

##### Query Data Tuple

In general, the queries in the CPS applications can be issued on multidimensional attributes, whereas the users’ interests on the multidimensional attributes tend to change constantly. For example, the query for transmission line conductor icing conditions in an ice disaster monitoring system requires multiple attributes: wind speed, temperature, humidity and other factors. The lower the temperature, the greater the humidity and the smaller the wind speed, and the easier the conductor freezes. To generate filter tuples for cutting nodes, this paper generates a query tuple, *qdata*, which is based on the original data normalization processing. The format of the tuple is as shown in Table 1.

**Table 1:** The format of *qdata*.

Node Label	Attribute Values	Maximum Value	Minimum Value	Attribute Space
*id*	*NLA(a* *,* *r)*	*max*	*min*	*reg*

In Table 1, *id* is the label of the node. *NLA*(a,r) are the attribute values of the original detected data after normalization. According to Equation (1), the sensor normalizes the detected data to [0,1] and unifies the trends in the sensing data values to the attribute values. The values have greater dominating ability if they are smaller. Therefore, it is convenient to compute the filter tuples that are used for cutting the nodes. In the formula, *a* is the initial attribute value, and* r* is the attribute query range that is used by the query when it is sent:

(1) $$NLA(a,r)=\left\{ \begin{array}{cc} {}[a-MIN(r)]/[MAX(r)-MIN(r)], & a's\text{ expectation value tends to small }\\ 1-[a-MIN(r)]/[MAX(r)-MIN(r)], & a's\text{ expectation value tends to big} \end{array}\right.$$

Suppose that *α*_i_ is the value of a tuple attribute and that it is normalized. Here, *n* is the number of tuple attributes, *max* is the maximum value in *NLA*(a,r), *min* is the minimum value in *NLA*(a,r), and *reg* is the attribute space that is the product of the attribute values in *NLA*(a,r). The smaller the value of *reg*, the smaller the tuple attribute values, which indicates that the tuple has more dominating ability. Equations (2)–(4) show the definitions of *max*, *min* and *reg*, as follows:

(2) $$max=MAX\{\alpha_{i}|\alpha_{i}\in NLA(a,r)\}$$

(3) $$min=MIN\{\alpha_{i}|\alpha_{i}\in NLA(a,r)\}$$

(4) $$reg=\overset{n}{\underset{1}{\prod }}\alpha_{i}$$

During the initialization of the query result collection, the node in the query routes judges its father node according to its node route table and transmits its own *qdata* to its father node. Then, the father node gathers the collected *qdata* to form a *TB_qdata* table.

##### Dynamic Filter Tuple

To cut the dominated nodes on the query route quickly and reduce the data transmission in the network, the paper proposes to generate the *filter_tuple* through a small amount of computation in the node table and to judge the dominated relation between the nodes accordingly. The tuple format is shown in Table 2.

**Table 2:** The format of *filter*_*tuple.*

Node Label	Minimum Value of *M*	Minimum Value of *N*	Minimum Value of *R*
*id*	*max_m*	*Min_m*	*reg_m*

Suppose that* M* is the set of* max* values of all of the tuples in table *TB_qdata*. *N* is the set of *min* values of all of the tuples. *R* is the set of *reg* values of all of the tuples. In a *filter*_*tuple*, *id* is the number of *filter*_*tuple*. Additionally, *max_m* is the minimum value of *M*, as follows:

(5) $$max\_m=MIN\{max_{i}|max_{i}\in M\}$$

*min_m* is the minimum value of *N*, as follows:

(6) $$min\_m=MIN\{min_{i}|min_{i}\in N\}$$

and *reg_m* is the minimum value of *R*, as follows:

(7) $$reg\_m=MIN\{reg_{i}|reg_{i}\in R\}$$

To generate the cutting tuple inside of a node, it sends *filter*_*tuple* to its father node during the query recovery initialization, and the *TB*_*Sfilters* table is composed of *filter*_*tuple* tuples, which belong to the father node and its children nodes.

#### 4.1.2. Initialization of the Query Result Collection Algorithm

During the initialization of the skyline query, a node will generate a *qdata* tuple after normalizing the collected original data. Each node sends its *qdata* tuple to its father node to enable the father node to create a *TB*_*qdata* table.

The node that owns the *TB_qdata* table generates *filter_tuple* after computing, and then the node gathers *filter_tuple* of both of its children nodes and itself to the *TB_Sfilters* table. Thus, this step is beneficial for the next step, which includes the cutting of dominated nodes and the fetching of the cutting tuple. The initialization algorithm of the query result collection is shown in Algorithm 1.


**Algorithm 1** Initialization of the Query Result Collection

**Input: **original node set *V* = {*S*_1_, *S*_2_, ..., *S*_n_}, the original data collected by node *S*_i_(i = 0, 1, ..., n)
**Output:***TB*_*qdata* table, *TB*_*Sfilters* table
1: **for** ∀ *S_i_* ∈ V **do {**
2: *S*_i_ normalizes the collected original data
3: Compute and add *max*, *min*, *reg* attributes to form *qdata*
4: *S*_i _sends its *qdata* tuple to its father node *S*_i+1_
5: *S*_i+1 _gathers the collected *qdata* to form the *TB*_*qdata* table
6: Fetch the minimum value of *max*, the minimum value of the attribute *min,* and the minimum value of the attribute* reg* of every tuple in the *S*_i+1_. *TB*_*qdata* table to form the tuple* S*_i+1_.*filter*_*tuple*
7: *S*_i+1_ sends each *filter*_*tuple* tuple to its father node *S*_i+2 _to form the *TB*_*Sfilters* table
8: **end for**


### 4.2. Node Cut

The existing WSN skyline query methods stress the domination relation among the data. The transmission consumption of large amounts of non-skyline data in the network is large. There exists a dominated relation among the nodes by analysing the detected data set of the nodes. In other words, one node can dominate all of the tuples of another sensor node. In the process of query result collection, the sensor finds the dominated node on the query route and cuts its computation by a small amount, which avoids frequent comparisons between the data, reduces the data communication traffic and reduces the network consumption.

#### 4.2.1. Node Domination Relation Theorems

Based on the basic concept of skyline query, the relations between nodes are defined as follows:

*Definition 2***.** The skyline nodes in the sensor network query and return all of the nodes that are not dominated by other nodes. Node *S*_i_ dominates node *S*_j_ (j ≠ i) if and only if a tuple in *S*_i_ can dominate all of the tuples in *S*_j_. It is also said that *S*_j_ is dominated by *S*_i_, which is denoted by *S*_i_ ≺ *S*_j_. Node *S*_j_ is not dominated by node *S*_i_ if and only if there exists at least one tuple in *S*_j_ that is not dominated by the tuple in *S*_i_, which is denoted by *S*_i_ ⊀ *S*_j_.

The following theorems exist between the attributes of the *TB_qdata* table and the attributes of the *TB*_*Sfilters* table. Before presenting the theorems, we define the symbols. Assume that a node can be represented by *S*_i_; let *V* = {*S*_1_, *S*_2_, …, *S*_n_} be the set of sensor nodes, *D* is the attribute set of the query data in one tuple, and *D*_k_ is an attribute of *D*. A tuple of *TB*_*qdata* in node *S_i_* is represented by *S_i_.t_m_*. Here, *t*(*D*_k_) is the value of *D*_k_ that belongs to *t*.

*Theorem 1***.** For ∃*t_m_*∈*S_i_.TB_qdata*, ∀ *D_k_*∈*D*, we have *S_i_.filter_tuple.max_m* ≥ *S_i_*. *t_m_*(*D_k_*).

*Proof:*

For ∃*D_d_*, *S_i_*.*t*(*D_d_*) = *S_i_*. *filter*_*tuple*.*max*_*m*.

In other words, *S_i_*.*t*(*D_d_*) = MAX{ *t_m_*(*D_k_*) | ∃*t_m_*∈*S_i_*.*TB*_*qdata*, ∀*D_k_*∈*D*}.

For ∃*t_m_*∈*S_i_.TB_qdata* and ∀*D_k_*∈*D*, we have *S_i_*.*t_m_*(*D_k_*) ≤*S_i_.t*(*D_d_*).

Thus, for ∃*t_m_*∈*S_i_*.*TB*_*qdata* and ∀*D_k_*∈*D*, we have *S_i_.filter_tuple.max_m* ≥*S_i_*.*t_m_*(*D_k_*).

Finish.

*Theorem 2.* For ∀*t_m_*∈*S_i_.TB_qdata* and ∀*D_k_*∈*D*, we have *S_i_*. *filter_tuple*.*min*_*m*≤*S_i_*.*t_m_*(*D_k_*).

*Proof:*

For ∃*D_d_*, *S_i_.t*(*D_d_*) = *S_i_.filter_tuple.min_m*.

In other words, *S_i_*.*t*(*D_d_*) = MIN{ *t_m_*(*D_k_*) | ∀*t_m_*∈*S_i_.TB_qdata*, ∀*D_k_*∈*D*}.

For ∀*t_m_*∈*S_i_.TB_qdata* and ∀*D_k_*∈*D*, we have *S_i_.t_m_*(*D_k_*)≥*S_i_.t*(*D_d_*) .

Thus, for ∀*t_m_*∈*S.TB_qdata* and ∀*D_k_*∈*D*, we have *S_i_*. *filter*_tu*p*le.*min*_*m* ≤*S_i_*.*t_m_*(*D_k_*). 

Finish.

*Theorem 3.* For ∃*S_i_* and *S_j_*∈*V*, if *S_i_*.*filter*_*tuple*.*min*_*m* < *S_j_*.*filter*_*tuple*.*min*_*m*, then we have *S_j_* ⊀ *S_i_*.

*Proof:*

∃*D_d_*, *D_d’_*∈*D*, ∃*S_i_*, *S_j _*∈ *V*, then

*S_i_*.*t*(*D_d_*) = *S_i_*.*filter*_*tuple*.*min*_*m*.

*S_j_*.*t*(*D_d’_*) = *S_j_*.*filter*_*tuple*.*min*_*m*.

By the known conditions, we have

*S_i_*.*filter*_*tuple*.*min*_*m* < *S_j_*.*filter*_*tuple*.*min*_*m*

Thus, *S_i_*.*t*(*D_d_*) < *S_j_*.*t*(*D_d’_*).

By Theorem 2, for ∀*t_m_*∈*S_j_*.*TB*_*qdata* and ∀*D_k_*∈*D*, then *S_j_*.*t*(*D_ d’_*) ≤*S_j_*.*t_m_*(*D_k_*).

Thus, we have *S_i_*.*t*(*D_d_*) < *S*_j_.*t_m_*(*D_k_*).

Thus, ∃*S_i_*, *S_j_*∈*V*, *S_i_*.*filter*_*tuple*.m*i*n_*m* < *S_j_*.*filter*_*tuple*.*min*_*m*.

In *S_i_*, there exists at least one tuple**that is not dominated by the tuple in *S_j_*. Then, we have *S_j_* ⊀ *S_i_*.

Finish.

*Theorem 4***.** For ∃*S_i_*, *S_j_*∈*V*, if *S_i_*.*filter*_*tuple*.*max*_*m* < *S_j_*.*filter*_*tuple*.*min*_*m*, then we have *S_i_*≺*S_j_*.

*Proof:*

∃*S_i_*, *S_j_*∈*V*.

∃*D_d_*, *S_i_*.*t*(*D_d_*) = *S_i_*.*filter*_*tuple*.*max*_*m*.

∃*D_d’_*, *S_j_*.*t*(*D_d’_*) = *S_j_*.*filter*_*tuple*.*min*_*m*.

By Theorem 1,

For ∃*t_m_*∈*S_i_*.*TB*_*qdata*, ∀*D_k_*∈*D*. we have *S_i_*.*t_m_*(*D_k_*) ≤ *S_i_*.*t*(*D_d_*).

By Theorem 2,

For ∀*t_m’_*∈*S_j_*.*TB*_*qdata* and ∀*D_k’_*∈*D*, we have *S_j_*.*t*(*D_d’_*) ≤ *S_j_*.*t_m’_*(*D_k’_*)

By the known conditions, we have

*S_i_*.*filter*_*tuple*.*max*_*m* < *S_j_*.*filter*_*tuple*.*min*_*m*.

Then, *S_i_*.*t*(*D_d_*) < *S_j_*.*t*(*D_d’_*).

We can move further, as follows:

For ∀*D_k_*∈*D*, ∀*D_k’_*∈*D*, ∃*t_m_*∈*S_i_*.*TB*_*qdata*; ∀*t_m’_*∈*S_j_*.*TB*_*qdata*, we have *S_i_*.*t_m_*(*D_k_*) < *S_j_*.*t_m’_*(*D_k’_*).

Thus, ∃*S_i_*, *S_j_*∈*V*, if *S_i_*.*filter*_*tuple*.*max*_*m* < *S_j._filter*_*tuple*.*min*_*m*, and then we have *S_i_*≺*S_j_*.

*Finish.*

#### 4.2.2. Node Cut Algorithm

Based on the above theorems, cutting a node means defining two sets in the node that owns the *TB*_*Sfilters* table, the certain node set *Q* and the uncertain node set *UQ*. The nodes in *Q* cannot be dominated by other nodes in *TB*_*Sfilters*. The nodes in *UQ* are the nodes for which the domination relationships among them cannot be determined. The original state of *UQ* is set to all of the nodes in the *TB*_*Sfilters* table. 

First, according to Theorem 3, the sensor analyses the node that owns the *TB*_*Sfilters* table; it determines the node that has the minimum *min* value in *UQ* and puts it into *Q*. Second, according to Theorem 4, the sensor judges the remaining nodes in *UQ* and deletes the nodes that are dominated by certain nodes out of *UQ*. The sensor repeatedly performs the above operations and eventually finalizes the set *Q*. In the phase of the skyline query result collection, we collect only the data from the nodes in *Q,* whereas the other nodes will be cut. The algorithm for the node cut is shown in Algorithm 2. Because the algorithm can cut the dominated nodes quickly, its calculation cost is small. Moreover, it can also reduce the consumption of the network communication and improve the efficiency of the query result collection.


**Algorithm 2** Node Cut

**Input:** The set *M* of nodes that own the *TB*_*Sfilters* table.
**Output:** The certain node set *Q*={*S_1_*, *S_2_*, ..., *S_m_*} and a new query route.
1: **for** ∀ *S_i_*∈*M*** do **// From bottom to top, the sensor conducts the algorithm to the node in *M* on the query route in turn 
2: **while **| *UQ* |>1 **do **// the number of nodes in *UQ* is greater than 1 
3: {
4: According to Theorem 3, the sensor finds the node *S_p_* that has the minimum min_m value in the *S_i_*.*TB*_*Sfilters* table of *UQ* and moves it from *UQ* to *Q*
5: According to Theorem 4, the sensor compares *S_p_*.*max_m* with ∀
*S_n_*.*min_m*(*S_n_* ∈ *UQ*), and the algorithm deletes the node that is dominated by the node *S_p_* in *UQ*
6: }
7: **if**
*S_q_*.*max*_*m*<*S_l_*.*min_m*
**then** //now, *S_q_* is the latest node put into *Q*, and *S_l_* is the last node in *UQ*
8: deletes *S_l_* from *UQ*
9: **else m**oves *S_l_* from *UQ* to *Q*
10: **end if**
11: The sensor deletes the nodes that do not belong to *Q* in the query route // generates a new query route
12: **end for**


### 4.3. Tuple Cut Inside the Node

#### 4.3.1. Extraction of Cutting Tuples

To address the tuple cut problem, this paper proposes to use the pre-cut tuple and the cutting tuple. First, the sensor creates a pre-cut tuple, *bcut_tuple*, according to the *TB*_*Sfilters* table of node *S*. The format of the *bcut*_*tuple* is shown in Table 3.

**Table 3:** The format of *bcut*_*tuple.*

Node Label	Smallest Attribute Space
*id*	*reg_ms*

Here, *reg_ms* is the smallest attribute space, and *reg_ms* = MIN{*reg_m*|*reg_m* ∈ *R*}, where *R* is the set of values of *reg_m*; id is the label of the node that corresponds to the tuple of the smallest value of *reg_s*. According to the value of id in *bcut*_*tuple*, the sensor finds out the *qdata* tuple in which the value of *reg* is equal to the value of *reg_ms* and its node has the same *id*. This tuple is the local cutting tuple, *cut*_*tuple*. Then, based on the local cutting tuple, the sensor conducts the tuple cut inside the nodes that are on the query route and whose child tree regards *S* as the root node. 

#### 4.3.2. Cutting the Tuples Inside the Node 

To improve the skyline query efficiency and reduce the data comparison and the amount of data transmission, this paper proposes to select a tuple that has the largest domination ability as the local cutting tuple in the query child tree whose root is the node in a bottom-up manner. The nodes in *Q* use this tuple to conduct a local cut to the query data of the node itself. After local cutting, the sensor sends the skyline data up to its father node and then to the root node. Finally, the root node collects the query results and sends them up to the base station. The algorithm for cutting the tuples and collecting the data inside the node is shown in Algorithm 3.


**Algorithm 3** Cutting the Tuples Inside the Node

**Input:** The set *M* of the nodes that own the *TB*_*Sfilters* table
**Output:** skyline data result
1: **for** ∀ *S_i_*∈*M*
**do** // From bottom to top, the sensor conducts the algorithm to the nodes in *M* on the query route in turn
2: create *bcut_tuple* by using *S_i_.TB_Sfilters*
3: **if**
*id*-value of *bcut_tuple* is equal to *S_i_.id*-value **then**
4: create *cut_tuple* by using *S_i_. TB_qdatas* and cut data in *S_i_.TB_qdatas*.
5: *S_i_* sends *cut_tuple* to its child nodes on the query route
6: **else**
7:* S_i_* sends *bcut_tuple* to the node *S_j_* with the same *id*. Here, *S_j_* is the child node of *S_i_*
8: create *cut_tuple* by using *S_j_.*
9: *S_j_* sends *cut_tuple* to *S_i_*
10: S_i_ sends *cut_tuple* to the other child nodes on the query route in turn, except for *S_j_*.
11: **end if**
12: the sensor received *cut_tuple* to conduct a local cut for the data in the *TB*_*qdata* table and deletes the non_skyline data*.*

13: Then, the sensor sends the skyline data up to its father node S_i_.
14: **if** S_i_ is one of the nodes of the query route, **then**
15: the skyline data received combine with the local data and send it to the father node
16: **else**
17: send the skyline data that were received to the father node 
18: **endif**
19:** end for**


### 4.4. E2SQ Query Analysis and Illustrations

The proposed energy-efficient skyline query algorithm for massively multidimensional sensing data adopts a node cut strategy to find the dominated nodes on the query path and to cut any sensing data sets of these nodes, through determining the domination relationship between the nodes while using a small amount of computation. Then, in the query subtrees that are rooted at the cluster nodes, the bottom-up approach is adopted to calculate and select a tuple as a local-cutting tuple, layer by layer. The last selected tuple has the greatest domination capacity. Additionally, the nodes in a certain set *Q* use these tuples to conduct a local cut for their own query data. This method avoids frequent data comparisons, reduces the amount of data communication, and reduces the energy consumption of the network. On the one hand, the E2SQ algorithm is to collect the data from the *n* nodes on the query path, and thus, the outer loop is *n*. On the other hand, the key part of the entire algorithm is the node cut and tuple cut inside the node strategies for each sub-tree for which the number of nodes in each sub-tree is much less than *n*, which results in the inner loop being a constant k. Thus, the time complexity of the algorithm E2Sky is O(kn), namely, O(n). Compared with the enormous energy consumption of the communications, the computational process consumes only a small amount of energy, and it can be ignored.

**Figure 3:**
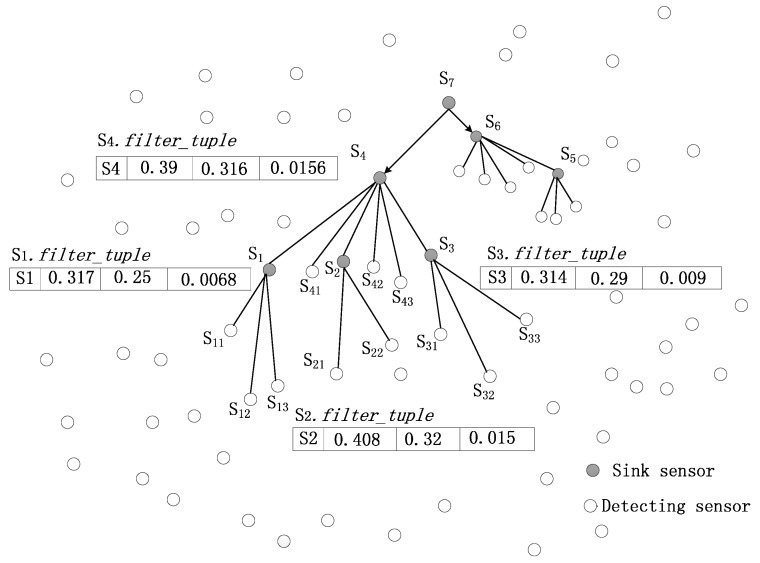
Initialization of the query recycling.

Taking the ice disaster monitoring system as an example, this paper adopts the energy-efficient Skyline query method to query the icing situation of a transmission line. After the query path is constructed based on physical windows, the node path table is generated on each node. Figure 3 shows the bottom of the query path. First, according to the Skyline query initialization algorithm, nodes *S_1_,S_2_,S_3_,S_4,_* respectively, collect *qdata* tuples of their own child nodes to generate *TB*_*qdata* tables, which are shown in Table 4, Table 5, Table 6 and Table 7, and they also calculate their own *filter_tuple* tuples. The *filter*_*tuples* of the nodes *S_1_*, *S_2_* and *S_3_* will be sent up to *S_4_*, respectively, and combined with the *filter*_*tuple* of S_4_ to form the *S_4_*.*TB*_*Sfilters* table, which is shown in Table 8.

**Table 4:** *S_1_*.*TB-qdatas* table.

Id	Temperature	Humidity	Wind_Speed	Wire_Angle	Max	Min	Reg
S11	0.317	0.25	0.31	0.313	0.317	0.25	0.0077
S12	0.328	0.26	0.27	0.2939	0.328	0.26	0.0068
S13	0.326	0.32	0.33	0.315	0.33	0.315	0.011

**Table 5:** *S_2_.TB-qdatas* table.

Id	Temperature	Humidity	Wind_Speed	Wire_Angle	Max	Min	Reg
S21	0.315	0.32	0.41	0.4	0.41	0.32	0.018
S22	0.346	0.33	0.33	0.408	0.408	0.33	0.015

**Table 6:** *S_3_.TB-qdatas* table.

Id	Temperature	Humidity	Wind_Speed	Wire_Angle	Max	Min	Reg
S31	0.303	0.32	0.33	0.353	0.353	0.303	0.011
S32	0.309	0.31	0.309	0.314	0.314	0.309	0.009
S33	0.343	0.29	0.41	0.383	0.41	0.29	0.016

**Table 7:** *S_4_.TB-qdatas* table.

Id	Temperature	Humidity	Wind_Speed	WIRE_ANGLE	Max	Min	Reg
S1	0.338	0.35	0.4	0.335	0.4	0.335	0.0159
S2	0.337	0.34	0.39	0.365	0.39	0.337	0.0163
S3	0.316	0.35	0.4	0.353	0.4	0.316	0.0156
S41	0.334	0.36	0.41	0.358	0.41	0.334	0.0176
S42	0.334	0.36	0.41	0.358	0.41	0.334	0.0176
S43	0.346	0.36	0.46	0.383	0.46	0.346	0.0219

**Table 8:** The *TB_Sfilters* table of *S_4_.*

Id	Max_M	Min_M	Reg_M
S1	0.317	0.25	0.0068
S2	0.408	0.32	0.015
S3	0.314	0.29	0.009
S4	0.39	0.316	0.0156

According to the node cut algorithm (see Algorithm 2), a specific node set *Q* can be defined for the node *S_4_*. In step 4 of Algorithm 2, *S_1_* will be moved to *Q*. In step 5, *S_2_* is the dominated node that should be deleted from the uncertain node set *UQ*, and now *S_3_* and *S_4_* remain in *UQ*. In step 4 of the algorithm, *S_3_* will be moved to *Q*. Now, *S_4_* is the only node in *UQ*. Through step 7 to step 9 of the algorithm, *S_3_* can dominate *S_4_*. Thus, *S_4_* will be deleted from *UQ*. Finally, the specific set *Q* = {*S_1_*, *S_3_*} is built, and *S_2_* and *S_4_* will be removed from the query route. 

Then, according to the algorithm for cutting tuples inside a node (see Algorithm 3), nodes *S_1_, S_3 _*execute the local cut algorithm to collect the Skyline data. By step 2 of Algorithm 3, a pre-cut tuple *bcut_tuple* (*S_1_*, 0.0068) is created, and it is sent down to its corresponding node *S_1_*. In step 8 of the algorithm, *S_1_* extracts *cut_tuple* (*S_1_*, 0.328, 0.26, 0.37, 0.2939, 0.328, 0.26, 0.0068) and sends it up to its father node *S_4_*. By step 10, *S_4_* sends *cut*_*tuple* down to node *S_3_*. Then, *S_1_* and *S_3_* cut their local tuples and upload their Skyline data, respectively.

## 5. Performance Evaluation

### 5.1. Experimental Setting

In this section, we verify the performance of our work by comparing E2Sky against three other methods in terms of the average communication cost, real skyline percentage and response time. These three methods are the TAG [26], MINMAX [21] and SkySensor [25]. Among these methods, TAG is used as the baseline algorithm, which uses a network computing algorithm. In our experiments, we vary the size of the sensor network from 100 m × 100 m to 500 m × 500 m with the steps of 100 m × 100 m. The default network size is 300 m × 300 m. We assume that all of the sensors are static and uniformly deployed and have a certain amount of memory, computing ability and transmission range. The node density of the sensor network is 1 node/10 m^2^. We further assume that the dimension of each tuple is three, that each attribute value is four bytes wide and that each sensor generates one tuple every time step. To evaluate the performance of E2Sky, we use the number of packets sent in the sensor network as a performance metric in the experiments. Each packet can contain data of up to 48 bytes. The simulated data are all generated by a standard dataset generator of the skyline query [27,28,29,30,31,32] in our experiments. There is no inherent relationship between different dimensional attributes of the detected data in the transmission line monitoring system application. In other words, our experiments consider only the performance of the independent data.

### 5.2. Average Communications Cost

In this experiment, we mainly test the performance of the average communications cost for E2Sky and the other three methods in terms of the network size, number of sensors and data dimensions. Here, the average communications cost refers to the average value of the communications cost to transfer the skyline data through executing the same experiment many times under the same parameter settings.

**Figure 4:**
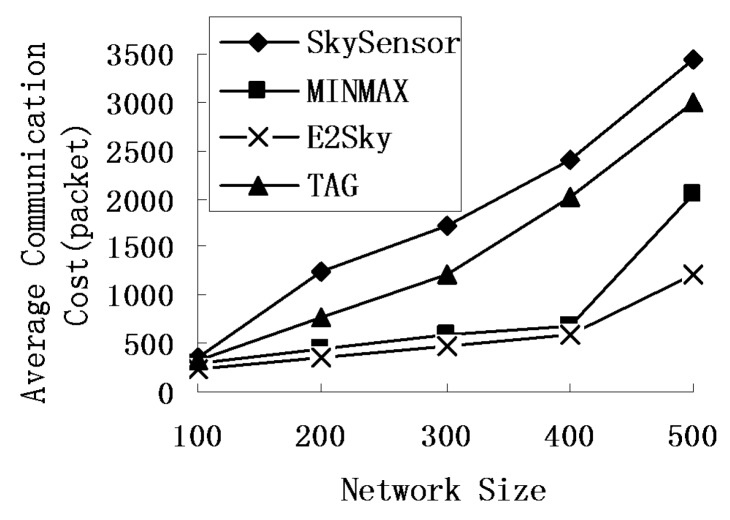
Average communications cost under different network sizes.

We study the performance of the average communications cost for E2Sky and the other three methods by varying the network size from 100 m × 100 m to 500 m × 500 m, and the node density of the sensor network is 1 node/10 m^2^. Figure 4 reveals that the average communications cost of those four methods increases with the size of the network, but E2Sky is the lowest. 

**Figure 5:**
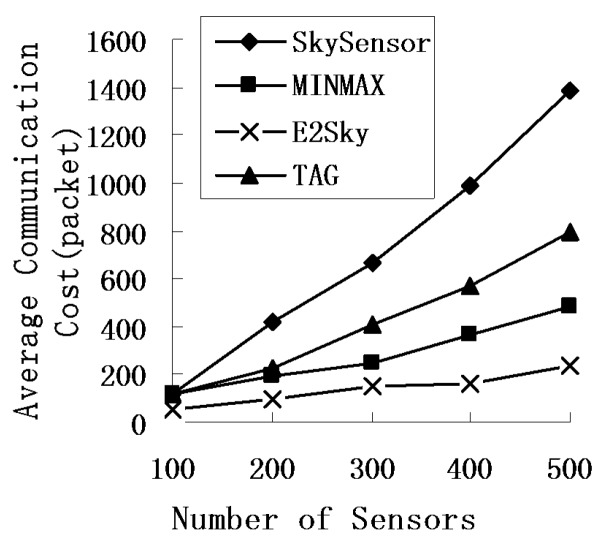
Average communications cost under a different number of sensors.

The reason is that when the network size grows large, the number of tuples that participate in the computation increases, which results in an increase in the skyline data, as well as the average communications cost. However, in the E2Sky algorithm, the data uploaded after each sensor’s cut are the real skyline results or only the minimal non-skyline data results, but those non-skyline data will be cut in the next node. Thus, compared with the other three algorithms, the average communications cost of E2Sky is the lowest. The performance of E2Sky is much less sensitive to the network size. The transmission cost for the detected data is transferred to a suitable cluster and is no longer shared by multiple queries. Therefore, the SkySensor is the highest. For the MINMAX algorithm using a filter, the average communications cost outperforms the baseline algorithm TAG. In the experiments of Figure 5, the network size is fixed at 300 m × 300 m, and the data dimension is 3. Figure 5 shows that with the increase in the number of sensors, the average communications cost of the four methods will increase. The reason is that an increase in the sensor nodes results in an increase in the data that participate in the computation and an increase in the skylines. The average communications cost of SkySensor, TAG and MINMAX grows rapidly at this point, but the growth in E2Sky is relatively slow. In the data collection process, the efficient dynamic filter generated by E2Sky needs a small amount of non-skyline data transmission in the network and a short transmission distance. Thus, the average communications cost of E2Sky is the lowest and the growth is relatively slow.

**Figure 6:**
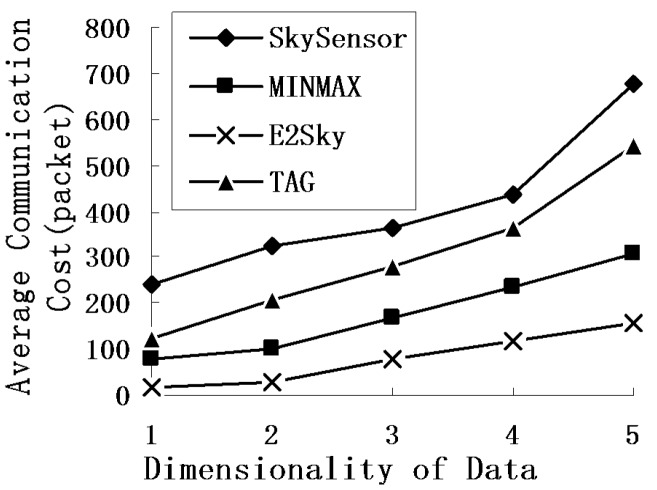
Average communications cost under dimensionality.

In the experiments in Figure 6, the network size is fixed at 300 m × 300 m, and the data dimension varies from 1 to 5. Figure 6 shows that as the data dimension increases, the average communications cost of those four methods becomes higher, also. The average communications cost of SkySensor, TAG and MINMAX is growing rapidly, but the growth in E2Sky is relatively slow. The reason is that the increase in the data dimensions leads to a decrease in the tuple domination probabilities, which enhances the skyline results and increases the average communications cost. When the data dimension increases, the efficient dynamic filter of E2Sky still has a good filtering effect, which makes the transmission distance of the non-skyline data very short or all of the non-skyline data are filtered out in the local sensor. At the same time, the E2Sky algorithm uses the cutting tuple to cut the detected data within each node. Therefore, the average communications cost of E2Sky is the lowest.

### 5.3. Accuracy of the Skyline Result

In this experiment, we mainly test the accuracy performance of the skyline result for E2Sky and the other three methods in terms of the network size, number of sensors and data dimensions. Here, the accuracy of the skyline result means the number of the real skyline data out of the total skyline result. We use the real skyline percentage to represent the accuracy of the skyline result.

**Figure 7:**
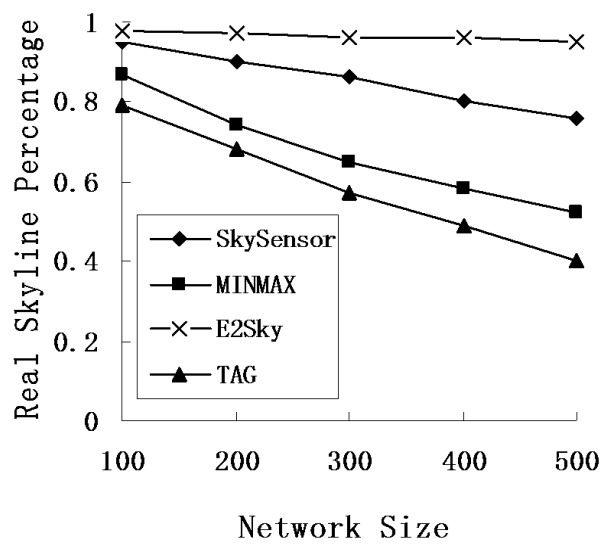
Real skyline percentage under different data and different network sizes.

We study the performance of the average communications cost for E2Sky and the other three methods by varying the network size from 100 m × 100 m to 500 m × 500 m, and the node density of the sensor network is 1 node/10 *m^2^*. Figure 7 demonstrates that the real skyline percentage of the other three methods, except for E2Sky, declines when the network size increases. When collecting the query results, E2Sky uses efficient dynamic filters to cut a large number of non-skyline results, and only small amounts of the non-skyline data must be transmitted in the network. Thus, the real skyline percentage of E2Sky outperforms the other three methods. SkySensor utilizes a cluster-based storage method to filter out parts of the non-skyline data from the clusters, progressively, in the pruning phase. However, in the gathering phase, there are still some non-skyline data that are returned to the query node. Although too much non-global skyline data remain, MINMAX, which generates its filter within each sensor, outperforms the baseline algorithm TAG. 

**Figure 8:**
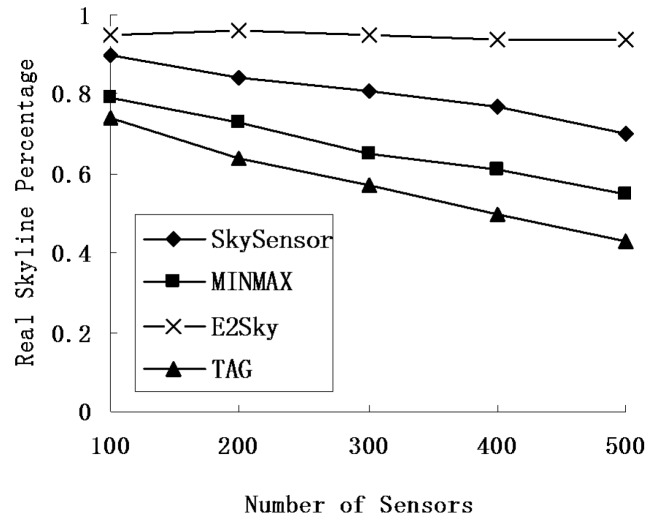
Real skyline percentage under a different number of sensors.

In the experiments shown in Figure 8, the network size is fixed at 300 m × 300 m, and the data dimension is 3. Figure 8 shows that the real skyline percentage of the three methods, except for E2Sky, decreases when the number of sensors increases. The reason is that with the increase in the number of sensor nodes, results on the depth of the referred routing tree will also be larger, and the uploaded skyline results will increase in the end. Thus, the real skyline percentage declines. The increasing number of sensors has no effect on the dynamic filter tuples and the local cut tuples of E2Sky. Therefore, E2Sky maintains a stable high percentage of the real skyline results.

**Figure 9:**
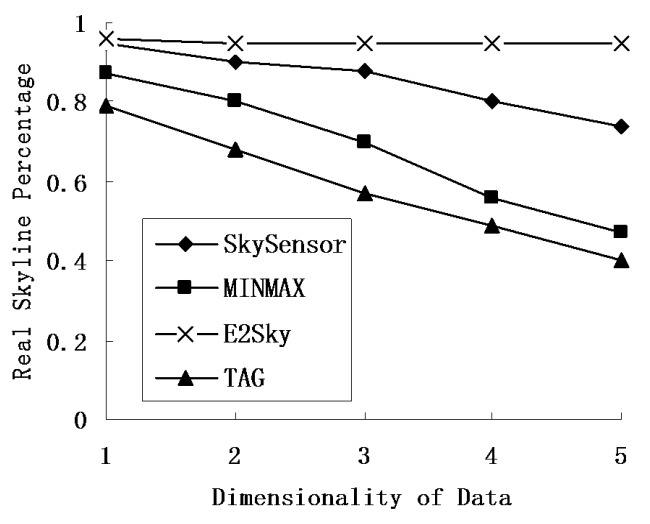
Real skyline percentage under different data dimensionality.

In the experiments of Figure 9, the network size is fixed at 300 m × 300 m, and the data dimension varies from 1 to 5. Figure 9 reveals that the real skyline percentage of the three methods, except for E2Sky, decreases when the data dimensions increases. The reason is that the increment of the data dimensions leads to a decrease in the probability of tuple domination, and more non-skyline data will be uploaded, which directly leads to a decline in the real skyline percentage. Although E2Sky takes a longer time to compute the dynamic filters, the filtering effect is still very good and causes only a small number of non-skyline results to remain. 

### 5.4. Response Time

In this experiment, we mainly tested the performance of the response time for E2Sky and the other three methods in terms of the network size, number of sensors and data dimensions. Here, we view the time for TAG to perform one network computing process as one time unit, and the other three methods use that as a reference. The response time of SkySensor refers to the quotient of the hops of the returned result hop and the depth of the routing tree of TAG.

**Figure 10:**
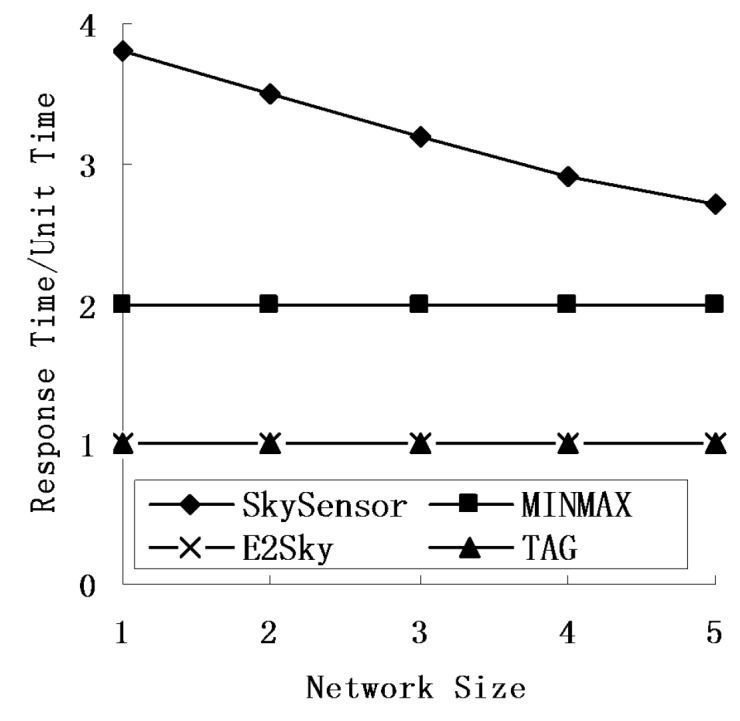
Response times under different network sizes.

**Figure 11:**
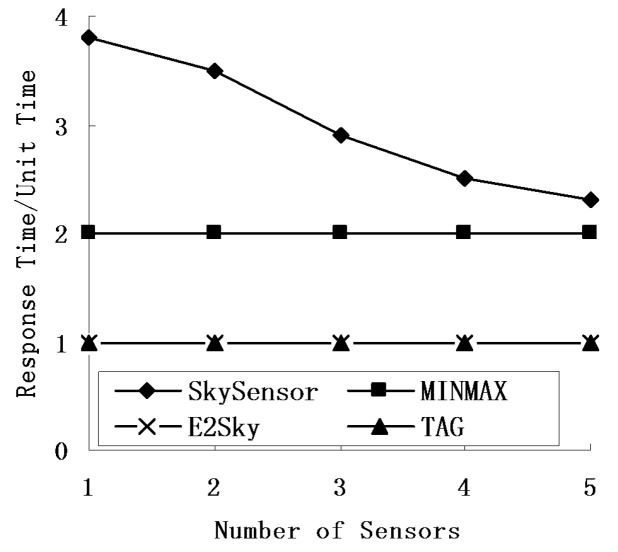
Response times under different numbers of sensors.

**Figure 12:**
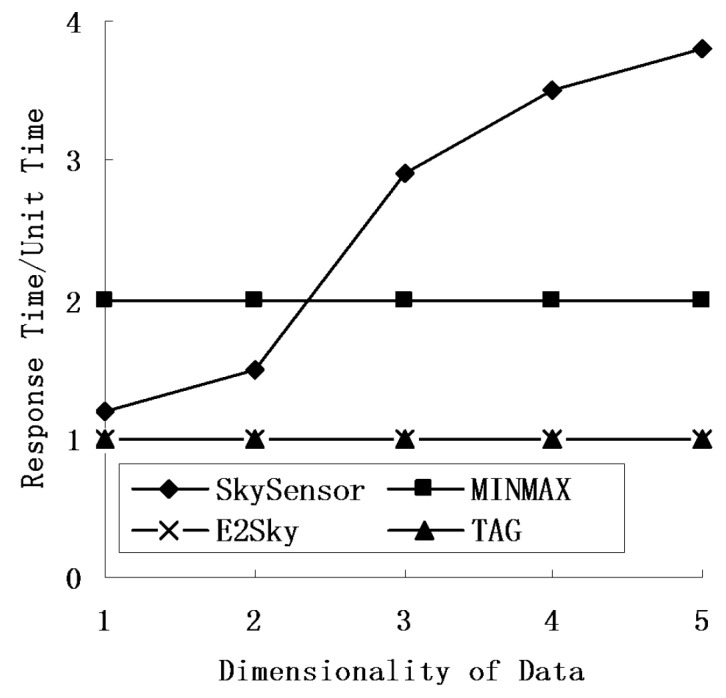
Response times under different data dimensionalities.

Figure 10, Figure 11 and Figure 12 show that regardless of the network size, the number of nodes or data dimensions increase, and only the response time of SkySensor is changing, whereas the other methods remain the same. E2Sky is the shortest, and it uses only one time unit. The reason is that E2Sky generates a dynamic filter tuple by a small computation when collecting the query results instead of issuing the query with a filter. The dynamic filter can cut the detected data of the dominated nodes rapidly to minimize the response time. Moreover, the response time of E2Sky is the same as TAG because E2Sky performs only one network computing process. With the increase in the network size, both the depth and the width of the referred routing trees increase. Hence, the response time of the SkySensor decreases slightly. 

In addition, the increase in the sensor nodes results in an increase of the depth of the referred routing tree. Thus, the response time of the SkySensor decreases progressively. Furthermore, because the number of clusters in a sensor network depends on the attributes of the detected data, when the data dimensions increase, the number of clusters in the network increases, also. This arrangement leads to an increase in the hop of the returned results, as well as an increase in the response time. In terms of the TAG algorithm, MINMAX is a two-phase algorithm, and the response time is two units of time.

### 5.5. Average Number of Accessed Sensor Nodes

In this experiment, we conduct the skyline query on the sensor nodes at the scale of 100 m × 100 m. The node density of the sensor network is 1 node/10 m^2^. In other words, the total number of sensors is 1000. The physical window of the query is the actual geographical range. Suppose that *R* is the percentage computed by the physical window size divided by the network size. This experiment mainly inspects the variety of the average number of accessed sensor nodes in each algorithm when the physical window of the query varies.

**Figure 13:**
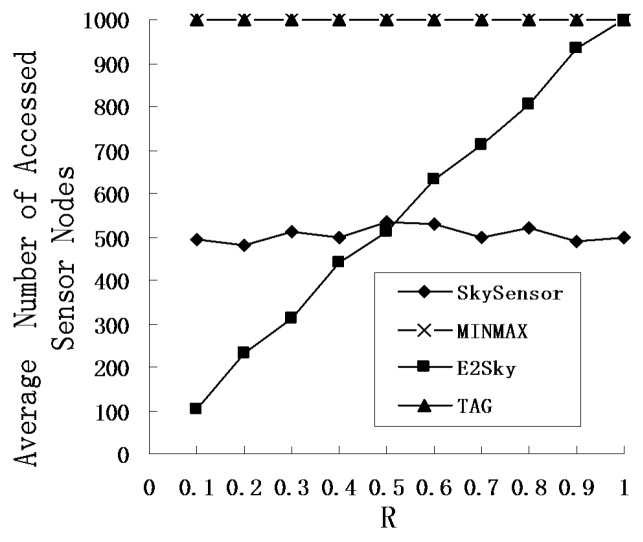
Average number of accessed sensor nodes under different values of *R*.

Figure 13 shows that only the average number of accessed sensor nodes in E2Sky increases when *R* increases and that the other three algorithms are essentially unchanged. The reason is that only the E2Sky algorithm can be based on the user requirements, and it executes the skyline query for the corresponding geographical location by using a specified query window when the query is sent. However, the other three algorithms must execute the skyline query in the entire network. As the E2Sky algorithm uses the storage structure of the routing tree, there will be some sensor nodes that belong to the neighbouring rooting tree or branch when the E2Sky algorithm is accessing the sensor nodes of the edge of the physical window. Hence, it must access the corresponding route of the routing tree or branch to obtain the detected data. The number of sensor nodes that are accessed in each query is always more than the number of sensor nodes that are contained in the physical window. 

The figure reveals that the E2Sky algorithm has a large advantage over the others when R is small. Because it needs to access only a few sensor nodes, the desired result will be regained. Even in the worst case, when the size of the physical window is equal to the network size, the average access number of the sensor nodes in E2Sky is equal to the algorithm of MINMAX and TAG, which also uses the storage structure of the routing tree. In addition, Skysensor is a method for a cluster-based architecture skyline query, and the detected data are distributed and stored in different clusters in the method. In the query processing, it can quickly prune the storage nodes that do not need to be visited in the storage clusters. In other words, in the best case, the algorithm needs to access only one sensor node in the cluster to obtain the skyline data. However, in the worst case, each node in the cluster will be accessed. Therefore, the average number of sensor nodes that are accessed in the SkySensor algorithm will be half of the total number of sensor nodes.

## 6. Conclusions

This paper proposes an energy-efficient skyline query for massively multidimensional sensing data. The proposed method uses a node cut strategy and generates a dynamic filter tuple by a small computation when collecting the query results instead of issuing the query with a filter. Thus, to judge the relationship among the nodes, it rapidly cuts the detected dominated nodes in the data set, and it reduces both the comparison frequency and the computing time. The efficient dynamic filter generated by the proposed strategy needs a small amount of non-skyline data transmission in the network and a short transmission distance. Then, by the cutting tuple strategy inside the node, the system can generate the local cut tuple with the child tree of the node itself and use it to cut the detected data within the nodes of the child tree. Hence, it can restrain the non-skyline data uploading further. Finally, the effectiveness of the algorithm is verified through synthetic data. The results show that the E2Sky algorithm can greatly reduce the data transmission in a WSN, shorten the response time of the algorithm and improve the efficiency of the query. When the scale of the WSN expands continuously, the method also scales well. It is shown that our algorithms can also be applied in a warning system for the possibility of failure or disaster, and it can improve the accuracy and timeliness of the warning. 
